# Protective Effect of Nopal Cactus (*Opuntia ficus-indica*) Seed Oil against Short-Term Lipopolysaccharides-Induced Inflammation and Peroxisomal Functions Dysregulation in Mouse Brain and Liver

**DOI:** 10.3390/ijms231911849

**Published:** 2022-10-06

**Authors:** Mounia Tahri-Joutey, Fatima-Ezzahra Saih, Riad El Kebbaj, Catherine Gondcaille, Joseph Vamecq, Norbert Latruffe, Gérard Lizard, Stéphane Savary, Boubker Nasser, Mustapha Cherkaoui-Malki, Pierre Andreoletti

**Affiliations:** 1 Laboratoire Biochimie, Neurosciences, Ressources Naturelles et Environnement, Faculté des Sciences et Techniques, Université Hassan I, BP577, Settat 26000, Morocco; 2 Laboratoire Bio-PeroxIL EA7270, University Bourgogne Franche-Comté, 6 Bd Gabriel, 21000 Dijon, France; 3 Laboratory of Health Sciences and Technologies, Higher Institute of Health Sciences, Hassan First University, Settat 26000, Morocco; 4 INSERM and HMNO, CBP, CHRU Lille, 59000 Lille and RADEME EA 7364, Faculté de Médecine, Université de Lille 2, 59045 Lille, France

**Keywords:** ACOX1, catalase, cactus seed oil, GPx, *Il-1β*, *Il-6*, *iNos*, lipopolysaccharides, peroxisome, SOD1

## Abstract

Exposure to endotoxins (lipopolysaccharides, LPS) may lead to a potent inflammatory cytokine response and a severe impairment of metabolism, causing tissue injury. The protective effect provided by cactus seed oil (CSO), from *Opuntia ficus-indica*, was evaluated against LPS-induced inflammation, dysregulation of peroxisomal antioxidant, and β-oxidation activities in the brain and the liver. In both tissues, a short-term LPS exposure increased the proinflammatory interleukine-1*β* (*Il-1β*), inducible Nitroxide synthase (*iNos)*, and Interleukine-6 (*Il-6*). In the brain, CSO action reduced only LPS-induced *iNos* expression, while in the liver, CSO attenuated mainly the hepatic *Il-1β* and *Il-6*. Regarding the peroxisomal antioxidative functions, CSO treatment (as Olive oil (OO) or Colza oil (CO) treatment) induced the hepatic peroxisomal *Cat* gene. Paradoxically, we showed that CSO, as well as OO or CO, treatment can timely induce catalase activity or prevent its induction by LPS, respectively, in both brain and liver tissues. On the other hand, CSO (as CO) pretreatment prevented the LPS-associated *Acox1* gene and activity decreases in the liver. Collectively, CSO showed efficient neuroprotective and hepato-protective effects against LPS, by maintaining the brain peroxisomal antioxidant enzyme activities of catalase and glutathione peroxidase, and by restoring hepatic peroxisomal antioxidant and β-oxidative capacities.

## 1. Introduction

Sepsis is associated with a high mortality rate and is defined by severe organ dysfunctions, necessitating urgent and intensive care [1,2]. Exposure to endotoxins (lipopolysaccharides, LPS), originating from bacterial membranes, may lead to an acute inflammatory cytokine response accompanied with a burst of reactive oxygen and a severe impairment of lipid metabolism, causing tissue injury [3,4,5]. Several studies have demonstrated that the destruction of the blood–brain barrier by LPS is involved in the development of several brain diseases, as for sepsis-associated encephalopathy [6]. The increased blood–brain barrier permeability by LPS has been correlated to tight and adherence junctions’ death and pericyte detachment [7,8], leading to the enhanced transport of pro-inflammatory cytokines [9,10]. The management of this acute sepsis syndrome depends on the body’s capacity to cope with the deleterious imbalance between the inflammatory cytokine burst, the important dysregulation of lipid metabolism, and the increased production of reactive oxygen species (ROS) [11,12]. Remarkably, peroxisome, as a cell compartment, englobes concomitantly both oxidase enzymes, generating ROS, and a set of enzymes able to metabolize H_2_O_2_ and other ROS species [5]. Among peroxisomal functions, the β-oxidation of very-long-chain fatty acids (VLCFA) is a critical pathway in the whole lipid metabolism. Indeed, VLCFA thioesters cross the peroxisomal membrane through ABC transporters called ABCD1 or ABCD2. Then, these fatty acyl-CoAs are handled by acyl-CoA oxidase 1 (ACOX1), the first and rate-limiting enzyme of peroxisomal β-oxidation. This reaction generates an enoyl-CoA and a hydrogen peroxide (H_2_O_2_) molecule, which is degraded by the peroxisomal catalase [11]. In addition, peroxisome contains a set of other ROS-scavenging enzymes, including epoxide hydrolase, glutathione peroxidase, peroxiredoxin I, peroxisomal membrane protein 20, and Cu-Zn superoxide dismutase (SOD) [13,14]. Importantly, the counterweight between peroxisomal β-oxidation and antioxidative activities notably contributes to cellular ROS homeostasis. Deficit affecting the peroxisome biogenesis or the specific peroxisomal activities may be linked with progressive neuronal demyelination, leading to the development of peroxisomal neurodegenerative diseases [15,16]. Several leukodystrophies are associated with the defect in the peroxisomal β-oxidation system, including a deficiency in VLCFA degradation. Accordingly, the defect in ABCD1 peroxisomal VLCFA transport is linked to X-linked adrenoleukodystrophy. The absence of fatty acyl-CoA β-oxidation is associated with ACOX1 deficiency disorder [11,15]. On the other hand, in rat liver, LPS exposure disturbs both fatty acid and phospholipid distribution in the peroxisomal membrane, as well as peroxisomal proteins expression [17]. Moreover, in rat C6 glial cells, LPS fully repressed ACOX1 expression and the oxidation of VLCFAs [17]. In addition, our group has shown the decreased expression of genes involved in hepatic peroxisomal fatty acid oxidation in LPS-treated mice [18].

We and other research teams have reported the chemical composition of cactus seed oil (CSO), which contains 62% linoleic acid (OO: 9.95% and CO: 19%), 21% oleic acid (OO: 76% and CO: 63%), and 12% palmitic acid (OO: 9% and CO: 4.5%), as particular fatty acids; 75.6 (mg/100 g oil) β-sitosterol (OO: 85 and CO: 44.5 mg/100 g oil), as a main phytosterol; and 68.4% γ-tocopherols (OO: 4.6% and CO: 69%) as major vitamin E components [19,20,21,22,23,24,25]. The analysis of CSO by Chougui et al. [20] has shown a highest content of polyphenols, flavonoids, and tannins in CSO than in the cactus fruit pulp. Furthermore, a recent detailed chemical analysis by Nounah et al. [21] revealed the presence of a large amount of phenolic compounds in CSO, which are known for their antioxidant and anti-inflammatory properties [26,27]. Like CSO, cactus peel oil is also enriched in essential fatty acids and liposoluble antioxidants [27]. Moreover, alkaloids, indicaxanthin, and various polyphenols and flavonoids have also been isolated from the cactus [28], as well as polysaccharides that are abundant in cladode extracts, harboring antidiabetic and antiglycation properties [27]. Our interest in CSO and argan oil revealed that two of their phytosterols (i.e., schottenol and spinasterol) activate the gene expression of two nuclear receptors, liver X receptors (LXR) α and β, and their target genes ABCA1 and ABCG1. This suggests that these two phytosterols play a protective role by modulating cholesterol metabolism in an LXR-dependent manner [29]. On the other hand, several reports have documented the health benefit of cactus *Ofi* compounds, showing their anticancer [30], antioxidant [27,31,32], antiproliferative [33], antiulcerogenic [27], hepatoprotective [34,35,36,37], and neuroprotective [31,38,39,40] effects.

Investigation on the protective effect of CSO on the brain and liver dysfunctions during sepsis has not been evaluated yet. Here, we investigate the short-term effect of LPS on brain and liver peroxisomal functions and inflammatory status in mice. The potential protective effect of CSO against LPS was compared to two common edible oils from olive (OO) and colza (CO). Furthermore, OO is well used in the Mediterranean diet, while CO is the most consumed oil in Europe [23]. The antioxidant capacity in the brain and liver was assessed by measuring the expression of proinflammatory genes as well as of the peroxisomal functions, including the fatty acid β-oxidation and antioxidants enzymes.

## 2. Results

In the present work, we attempted to evaluate the protective effect of CSO on the brain and liver in a short-term 4 h post-LPS injection. The effects of CSO and two other standard edible oils, olive and colza, on peroxisomal antioxidative and β-oxidative functions and cellular inflammation markers in both brain and liver were compared. Two groups of mice received each for 28 days a standard chow supplemented or not with 6% (*w*/*w*) of one of the three compared oils (CSO, OO, or CO). Four hours before euthanasia, mice from the first group received an injection of 100 µg LPS via tail vein, while the control group received instead, an injection of PBS.

### 2.1. Inflammatory Biomarkers

**Figure 1:**
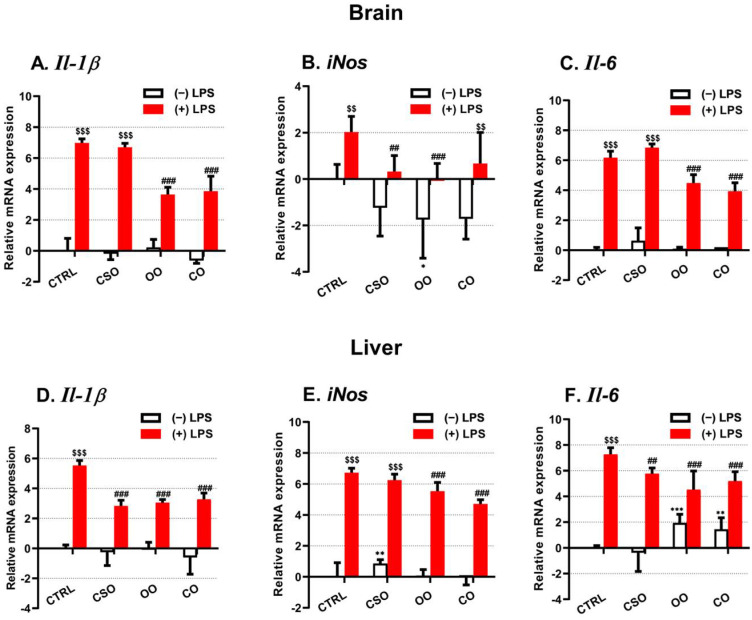
Effect of cactus seed oil, olive oil, or colza oil treatment on gene expression of the proinflammatory markers Il-1β (**A**,**D**), iNos (**B**,**E**), and Il-6 (**C**,**F**), in the brain and liver, respectively. Male C57BL/6 mice received for 28 days a standard diet (control (CTRL)), a diet enriched with 6% (*w*/*w*) CSO, OO, or CO, and intravenous injection of LPS (100 µg) four hours antemortem. First, total RNA was isolated from mice brains or livers, and then the expression level of genes of interest was quantified by real-time RT-qPCR. All values are means ± SD (n = 4–6), statistical significance of higher mean signal (*** *p* ≤ 0.001. ** *p* ≤ 0.01.) compared to control, (### *p* ≤ 0.01. ## *p* ≤ 0.01) compared to LPS, and ($$$ *p* ≤ 0.001. $$ *p* ≤ 0.01) compared to the different treatments with or without LPS administration. Statistics were executed using two-way ANOVA followed by Tukey test for multiple comparisons.

**Figure 2:**
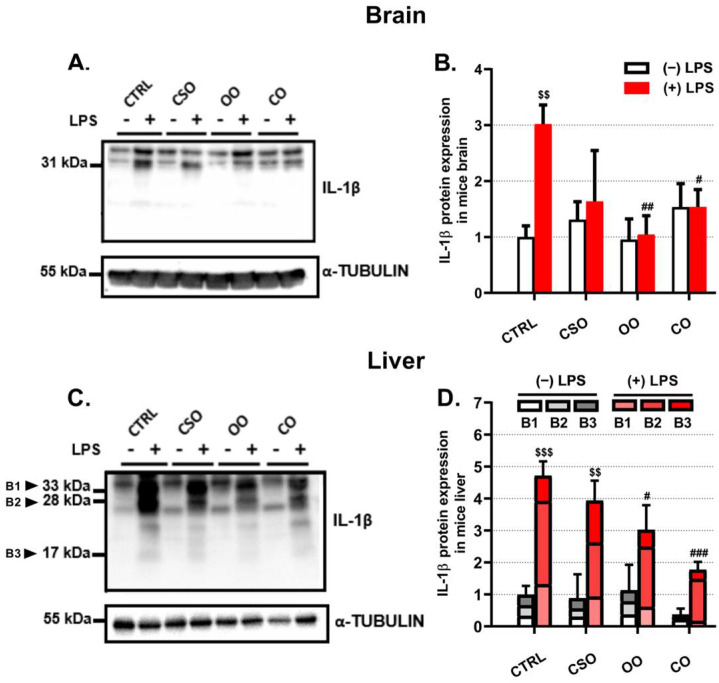
Effect of cactus seed oil, olive oil, or colza oil treatment on protein expression of the proinflammatory marker Il-1β (**A**–**D**), in the brain (**A**,**B**) and liver (**C**,**D**), respectively. Male C57BL/6 mice received for 28 days a standard diet (control (CTRL)), a diet enriched with 6% (*w*/*w*) CSO, OO, or CO, and intravenous injection of LPS (100 µg) four hours antemortem. Brain and liver homogenates were separated in PAGE-SDS electrophoresis and subjected to immunoblotting as described in Material and Methods section. Immunoblots were performed in triplicate and here we showed a representative blot. The three processed Il-1β bands (B1: 33 kDa; B2: 28 kDa; B3:17 kDa) intensities were analyzed by densitometry and standardized to α-tubulin (55 kDa) expression level in brain (**B**) and in liver (**D**). All values are means ± SD (n = 3) of 3 independent replicates. Statistical significance of higher mean signal strength compared to control, (### *p* ≤ 0.01. ## *p* ≤ 0.01. # *p* ≤ 0.05) compared to LPS, and ($$$ *p* ≤ 0.001. $$ *p* ≤ 0.01) compared to the different treatments with or without LPS administration. Statistics were executed using two-way ANOVA followed by Tukey test for multiple comparisons.

**Figure 3:**
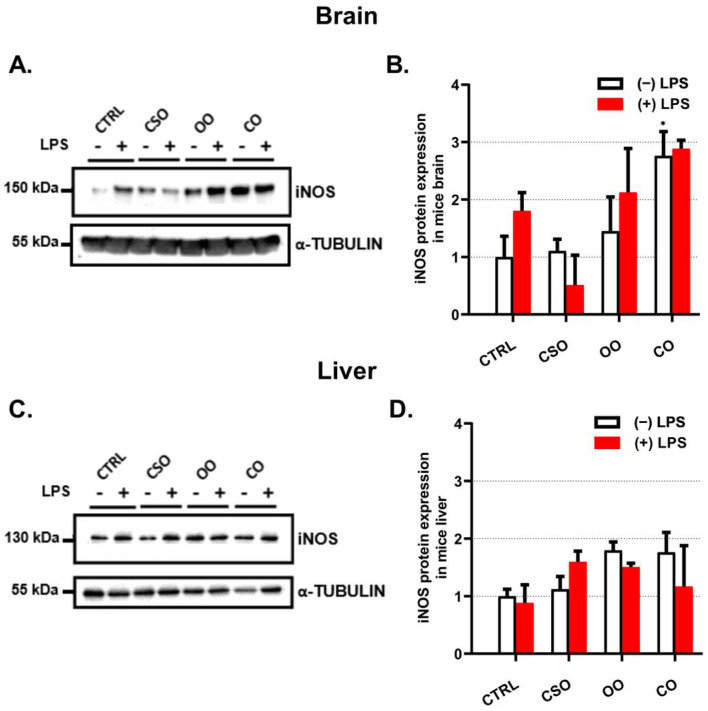
Effect of cactus seed oil, olive oil, or colza oil treatment on protein expression of the iNOS in the brain (**A**) and liver (**C**), respectively. Male C57BL/6 mice received for 28 days a standard diet (control (CTRL)), a diet enriched with 6% (*w*/*w*) CSO, OO, or CO, and intravenous injection of LPS (100 µg) four hours antemortem. Brain and liver homogenates were separated in PAGE-SDS electrophoresis and subjected to immunoblotting as described in Material and Methods section. Immunoblots were performed in triplicate and here we showed a representative blot. The iNOS 130 kDa band intensities were analyzed by densitometry and standardized to α-tubulin (55 kDa) expression level in brain (**B**) and in liver (**D**). All values are means ± SD (n = 3) of 3 independent replicates. Statistical significance of higher mean signal strength (* *p* ≤ 0.05) compared to control, compared to LPS, and compared to the different treatments with or without LPS administration. Statistics were executed using two-way ANOVA followed by Tukey test for multiple comparisons.

The transcript levels of the proinflammatory marker Il-1β were evaluated in the brain and the liver (Figure 1A,D). Of note, in both brain and liver tissues the basal expression level of Il-1β was not affected by any oil treatment alone (Figure 1A,D), while the LPS treatment increased significantly both brain and liver Il-1β mRNA levels (Figure 1A,D). This LPS transcriptional response was partially attenuated by OO or CO pretreatment (Figure 1A,D). However, the CSO pretreatment had an attenuating effect only in the brain of LPS-treated mice, but not in the liver (Figure 1A,D). At the protein level, IL-1β was largely induced by LPS in the brain and the liver (Figure 2A,C). However, the processing of the pro-IL-1β form (33 kDa) to its active forms (28 and 17 kDa) was detectable only in the liver (Figure 2C). Thus, CSO treatment showed significant attenuated hepatic levels of pro-IL-1β and its processed active forms (Figure 2C,D). iNos gene expression was shown to be upregulated in an IL-1β-dependent manner [41]. CSO administration had an opposite effect on iNos expression between the brain and liver, showing a tendency to decrease brain iNos expression and a significant increase in the hepatic iNos mRNA level (Figure 1B,E). The OO treatment resulted in a significant downregulation of iNos transcript levels only in the brain (Figure 1B,E). LPS induced the iNos mRNA expression in the liver and at a lesser extent in the brain. Nonetheless, only CSO or OO pretreatment was able to abrogate, specifically in the brain, this LPS-dependent induction (Figure 1B,E). The brain and liver iNOS protein expression showed no significant variations regarding LPS and/or oils treatments (Figure 3A–D). With respect to oil treatments, we did not observed the modification of Il-6 expression in the brain (Figure 1C). However, we noticed a slight but significant increase of Il-6 mRNA level in the liver upon OO or CO treatment (Figure 1F). Our data showed that CSO treatment regulated the expression of Il-1β, iNos, and Il-6 in a tissue-dependent manner.

**Figure 4:**
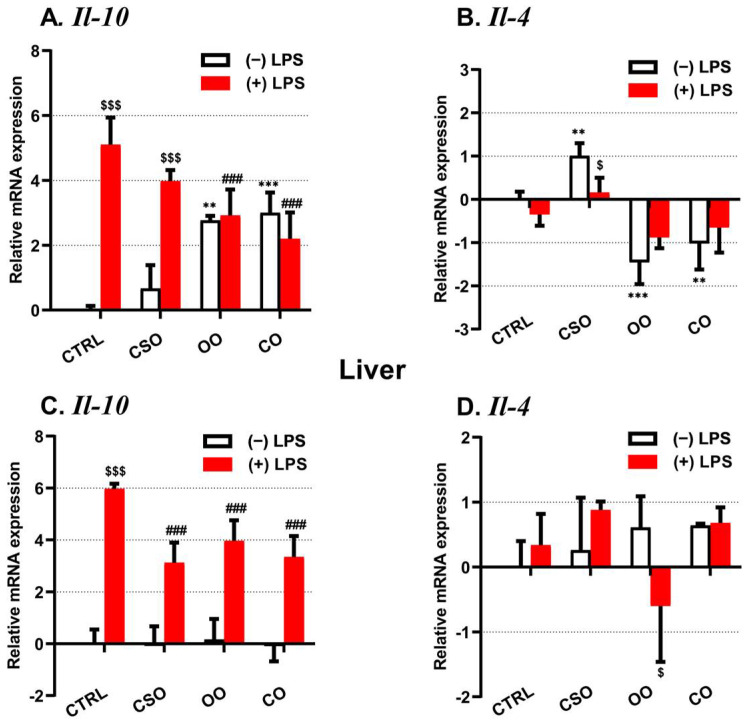
Effect of cactus seed oil, olive oil, or colza oil treatment on gene expression of the anti-inflammatory markers Il-10 (**A**,**C**) and Il-4 (**B**,**D**), in the brain and liver, respectively. Male C57BL/6 mice received for 28 days a standard diet (control (CTRL)), a diet enriched with 6% (*w*/*w*) CSO, OO, or CO, and intravenous injection of LPS (100 µg) four hours antemortem. First, total RNA was isolated from mice brains or livers, and then the expression level of genes of interest was quantified by real-time RT-qPCR. All values are means ± SD (n = 4–6), statistical significance of higher mean signal (*** *p* ≤ 0.001. ** *p* ≤ 0.01) compared to control, (### *p* ≤ 0.01) compared to LPS, and ($$$ *p* ≤ 0.001. $ *p* ≤ 0.05) compared to the different treatments with or without LPS administration. Statistics were executed using two-way ANOVA followed by Tukey test for multiple comparisons.

On the other hand, the expression of the anti-inflammatory Il-10 and Il-4 genes has been evaluated. Only the Il-10 mRNA expression has been strongly induced by LPS in the brain and the liver tissues (Figure 4A,C). Both OO or CO supplementation showed an opposite effect, inducing Il-10 and decreasing Il-4 mRNA levels in the brain (Figure 4A,B), while CSO treatment induced only the brain Il-4 expression (Figure 4B). CSO (as OO or CO) attenuated specifically the LPS-dependent induction of Il-10 gene expression in the brain and the liver (Figure 4A,C).

### 2.2. Brain and Liver Gene Expression of Peroxisomal Protein-Encoding Genes

**Figure 5:**
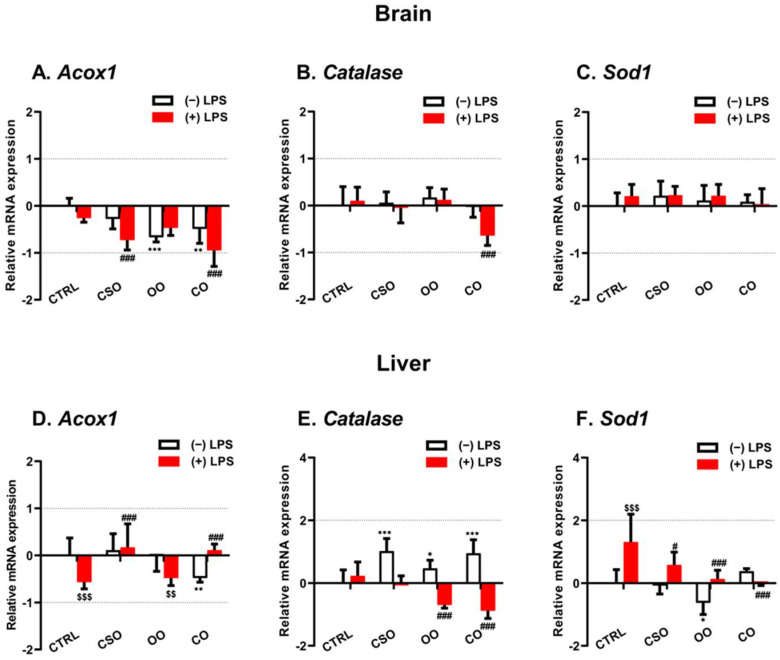
Effect of cactus seed oil, olive oil, or colza oil treatment on gene expression of Acox1 (**A**,**D**), Cat (**B**,**E**), and Sod1 (**C**,**F**) in brain and liver, respectively. Male C57BL/6 mice received for 28 days a standard diet (control (CTRL)), a diet enriched with 6% (*w*/*w*) CSO, OO, or CO, and intravenous injection of LPS (100 µg) four hours antemortem. First, total RNA was isolated from mice brains and livers, and then the expression level of genes of interest was quantified by real-time RT-qPCR. All values are means ± SD (n = 3), statistical significance of higher mean signal (*** *p* ≤ 0.001. ** *p* ≤ 0.01. * *p* ≤ 0.05) compared to control, (### *p* ≤ 0.01, # *p* ≤ 0.05) compared to LPS, and ($$$ *p* ≤ 0.001. $$ *p* ≤ 0.01) compared to the different treatments with or without LPS administration. Statistics were executed using two-way ANOVA followed by Tukey test for multiple comparisons.

Next, we evaluated LPS and oil treatment effects on the expression of three peroxisomal genes encoding ACOX1, CAT, and SOD1. The brain Acox1 mRNA expression did not show significant changes after CSO administration alone, while a significant decrease was observed following OO or CO treatment (Figure 5A). The short-term LPS injection had no effect on the brain Acox1 mRNA expression. However, in the CSO-LPS or CO-LPS treated mice, the level of brain Acox1 transcripts was significantly decreased (Figure 5A). In the liver, only CO treatment revealed a significant downregulation of Acox1 mRNA (Figure 5D). In response to LPS administration, the liver Acox1 mRNA level was significantly diminished in the LPS group compared with the control group (Figure 5D). Either CSO or CO pretreatment showed a protective effect against LPS injection in the liver (Figure 2D).

In the brain, Cat mRNA expression was not changed by any oil treatment in the absence or presence of LPS administration, excluding CO-LPS, which showed a significant decreasing level (Figure 5B). However, in the liver, a significant increase was shown in CSO-treated mice, or after treatment with OO or CO. As in the brain, LPS administration had no effect on Cat mRNA expression, while OO- or CO-pretreated mice receiving LPS revealed a downregulation of Cat mRNA level (Figure 5E). The brain Sod1 mRNA level was not induced by LPS injection, and no other changes were noted in all oil-pretreated mice receiving LPS or not (Figure 5C). However, in the liver, only OO treatment showed a significant decrease in Sod1 transcripts level, while LPS-induced expression of Sod1 was attenuated by CSO, OO, or CO pretreatment (Figure 5F).

### 2.3. Brain and Liver Expressions of Peroxisomal Proteins

ACOX1 is the first rate-limiting enzyme of peroxisomal fatty acyl-CoA β-oxidation, which produces enoyl-CoA and H_2_O_2_ as by-product of this reaction. CAT is a major peroxisomal protein, which degrades hydrogen peroxide. ACOX1 and CAT protein contents were assessed by immunoblotting.

#### 2.3.1. Catalase Protein Expression

**Figure 6:**
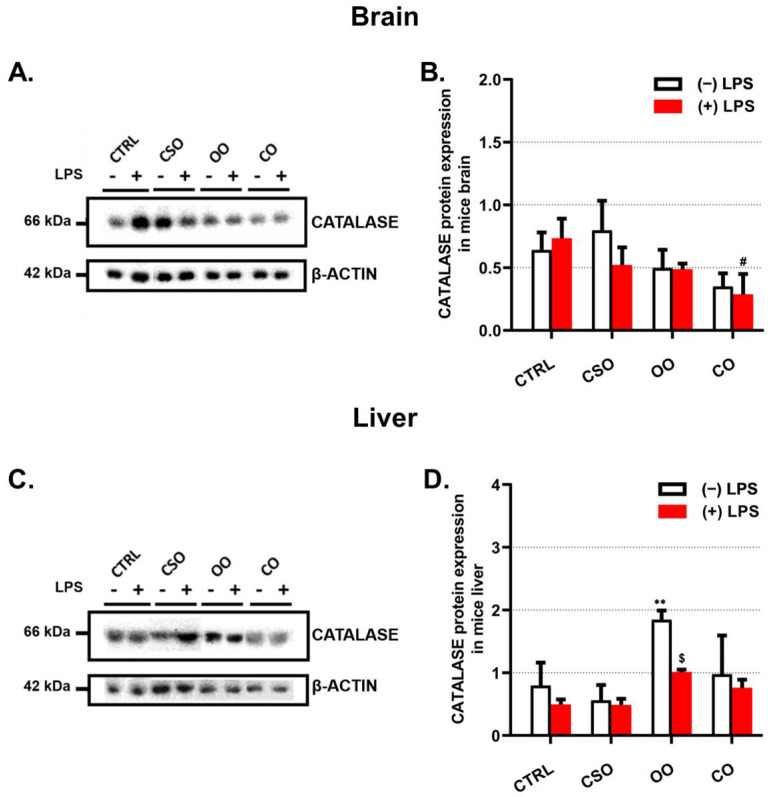
Effect of cactus seed oil, olive oil, or colza oil treatment on the expression of peroxisomal CAT in brain (**A**) and in liver (**C**). Male C57BL/6 mice received for 28 days a standard diet (control (CTRL)), a diet enriched with 6% (*w*/*w*) CSO, OO, or CO, and intravenous injection of LPS (100 μg) four hours antemortem. Brain and liver homogenates were separated in PAGE-SDS electrophoresis and subjected to immunoblotting as described in Material and Methods section. Immunoblots were performed in triplicate and here we showed a representative blot. The CAT 66 kDa band intensities were analyzed by densitometry and standardized to β-actine (42 kDa) expression level in brain (**B**) and in liver (**D**). All values are means ± SD (n = 3) of 6 independent replicates for CTRL, LPS, CSO, and CSO+LPS, and 3 replicates for OO, OO+LPS, CO, and CO+LPS; statistical significance of higher mean signal strength (** *p* ≤ 0.01) compared to control, (# *p* ≤ 0.05) compared to LPS, and ($ *p* ≤ 0.05) compared to the different treatments with or without LPS administration. Statistics were executed using two-way ANOVA followed by Tukey test for multiple comparisons.

In the brain, CAT protein levels showed no significant change after oil treatment or LPS administration. However, CO pretreatment in the absence or the presence of LPS diminished the catalase content (Figure 6A,B). Remarkably, in the liver, OO treatment induced significantly hepatic CAT levels (Figure 6C,D), while the CAT amount was reduced by LPS administration (Figure 6C,D).

#### 2.3.2. ACOX1 Protein Expression

**Figure 7:**
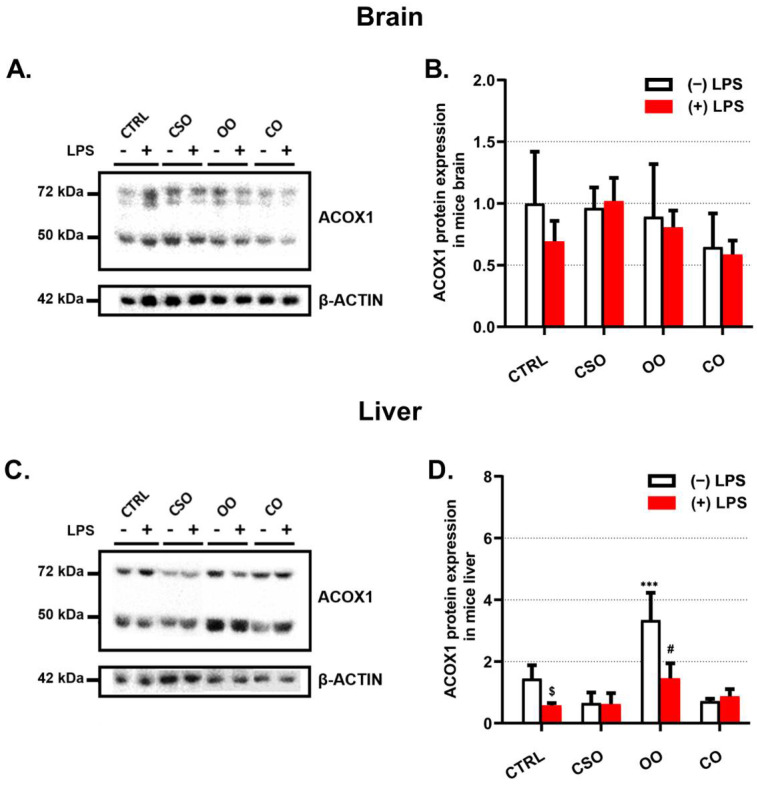
Effect of cactus seed oil, olive oil, or colza oil treatment on the expression of peroxisomal ACOX1 in brain (**A**) and in liver (**C**). Male C57BL/6 mice received for 28 days a standard diet (control (CTRL)), a diet enriched with 6% (*w*/*w*) CSO, OO, or CO, and intravenous injection of LPS (100 μg) four hours antemortem. Brain and liver homogenates were separated in PAGE-SDS electrophoresis and subjected to immunoblotting as described in Material and Methods section. Immunoblots were performed in triplicate and here we showed a representative blot. The ACOX1 72 and 52 kDa band intensities were analyzed by densitometry and standardized to β-actine (42 kDa) expression level in brain (**B**) and in liver (**D**). All values are means ± SD (n = 3) of 6 independent replicates for CTRL, LPS, CSO, and CSO+LPS, and 3 replicates for OO, OO+LPS, CO, and CO+LPS; statistical significance of higher mean signal strength (*** *p* ≤ 0.001) compared to control, (# *p* ≤ 0.05) compared to LPS, and ($ *p* ≤ 0.05) compared to the different treatments with or without LPS administration. Statistics were executed using two-way ANOVA followed by Tukey test for multiple comparisons.

ACOX1, a 72 kDa polypeptide, is imported into peroxisomes and incompletely processed into 51 and 21 kDa protein products. ACOX1 functions as a dimer, composed of only 72 kDa polypeptides or a combination of 72, 51, and 21 kDa polypeptides [42]. Regarding the brain tissue, LPS showed a non-significant decrease in the expression of ACOX1 content, while the CSO pretreatment restored the expression of ACOX1 to the same level as the control one (Figure 7A,B). Neither OO nor CO significantly changed the content of brain ACOX1 when compared to the control (Figure 7A,B). On the other hand, the hepatic ACOX1 level was reduced by LPS as well as by CSO treatment alone (Figure 7C,D).

As for CAT, OO treatment increased liver ACOX1 content and similarly in OO-LPS mice when compared to their corresponding controls (Figure 7C,D).

### 2.4. Brain and Liver Peroxisomal Enzymes Activities

**Figure 8:**
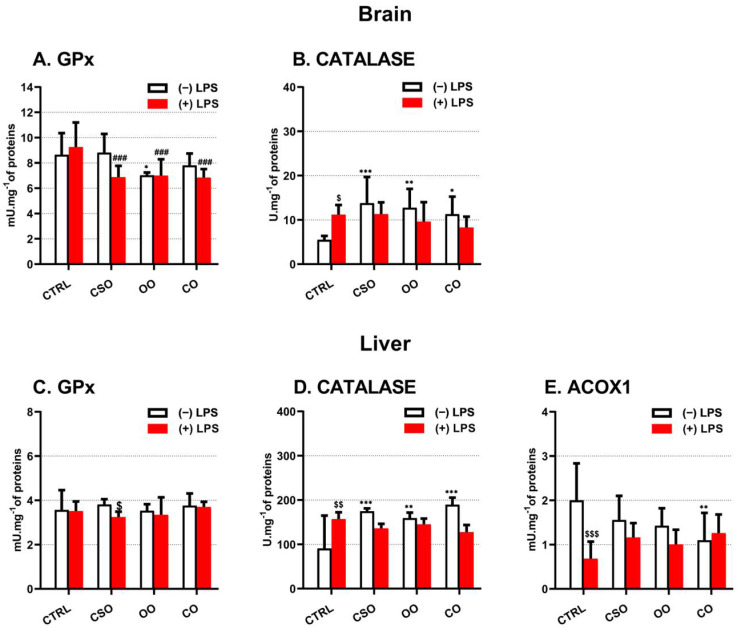
Effect of cactus seed oil, olive oil, or colza oil treatment on the peroxisomal enzymes activities of brain and liver GPx (**A**,**C**) and CAT (**B**,**D**), respectively, and on liver ACOX1 (**E**). C57BL/6 mice received for 28 days a standard diet (control (CTRL)), a diet enriched with 6% (*w*/*w*) CSO, OO, or CO, and intravenous injection of LPS (100 μg) four hours antemortem. Brain and liver homogenates were prepared as described in Material and methods section. Results are expressed in (UI.mg^−1^ = one μmol of substrate transformed/minute/mg of proteins). All values are means ± SD (n = 6), statistical significance of higher mean signal strength (*** *p* ≤ 0.001. ** *p* ≤ 0.01. * *p* ≤ 0.05) compared to control, (### *p* ≤ 0.01) compared to LPS, and ($$$ *p* ≤ 0.001 and $$ *p* ≤ 0.01, $ *p* ≤ 0.05) compared to the different treatments with or without LPS administration. Statistics were executed using two-way ANOVA followed by Tukey test for multiple comparisons.

The catalytic activities of two peroxisomal antioxidant enzymes, GPX and CAT, were measured in both the brain and liver from the different groups of mice. In both brain and liver tissues, LPS had no effect on the GPx activity level, while pretreatment with CSO reduced GPx activity in LPS-CSO mice (Figure 8A,C). Similar results were obtained with OO or CO pretreatment in the mice brain (Figure 8A). Interestingly, in the brain, the CAT activity was increased whatever the oil treatment. LPS significantly increased the activity of CAT and pretreatment with oil did not change the LPS effect (Figure 8B). By contrast, in the liver, LPS induced catalase activity (Figure 8D). The administration of LPS to oil-pretreated mice did not attenuate the induced CAT activity, when compared to the LPS-treated control. However, CSO pretreatment significantly increased the hepatic CAT activity (Figure 8D). Despite the sensitivity of the fluorometric method, the ACOX1 enzymatic activity measurement in brain homogenates was below the detectable threshold. Nonetheless, the measurements of liver ACOX activity reveal a negative effect of LPS administration (Figure 8E). Neither CSO or OO oil was able to significantly affect the level of hepatic ACOX1 activity, except CO oil treatment that reduced the activity level of ACOX1. By contrast, when mice were pretreated with CSO, OO, or CO, we observed almost a restoration of ACOX1 activity to its control level (Figure 5E), suggesting that CSO oil possesses similar properties as olive oil.

## 3. Discussion

A recent report from the Food and Agriculture Organization of the United Nations highlighted the growing interest across the word in cactus pear (Opuntia ficus-indica: Ofi) for its multiple purposes [38]. The interest in Ofi keeps growing not only because of its exceptional adaptation to arid and semi-arid climates in tropical and subtropical regions and its characteristics that provide resilience to restore degraded land, but also because Ofi is now considered as a source of functional foods, which can provide phytochemicals of nutraceutical interest [14,39]. Previous studies have collected compelling evidence of the protective properties of CSO from Ofi and particularly from other Opuntia species against chronic diseases such as cancer and diabetes [19,28,40]. However, the potential protective effect of CSO against LPS-induced peroxisome dysfunction and inflammation in brain and liver tissues has not been investigated so far. Here, the present study affords evidence that a CSO-supplemented diet has a protective effect against the deleterious endotoxic LPS shock in mouse brain and liver, regarding the inflammatory status and peroxisomal antioxidative and fatty acid β-oxidation pathways.

The evaluation of the inflammatory status after a four-hour LPS injection showed a strong increase in the proinflammatory *Il-1β*, *iNos*, and *Il-6* gene expressions in both the brain and the liver tissues. In addition, we observed in the liver that the LPS-induced expression of pro-IL-1β (33 kDa) was processed to its 17 kDa active form. Here, we showed that CSO prevented LPS-induced inflammation with differential responses between brain and liver. In the brain, CSO action reduced only LPS-induced *iNos* gene expression. However, the short treatment by LPS (i.e., 4 h) revealed an absence of significative changes in iNOS protein expression. The iNOS protein expression was reported to be a late event not observed before 12 h of LPS treatment [43]. In the liver, CSO treatment attenuated mainly *Il-1β* and *Il-6* gene expression and proIL-1β protein expression as well as its processed active forms, particularly the 17 kDa form [44]. Previously, Lee et al. [45] reported that the ethanolic extract of *Ofi var. saboten* stem reduced *iNos* expression in the LPS-activated murine brain microglial BV-2 cell line. In addition, the *Ofi* extract also inhibited the degradation of IκB-α in BV-2 cells, resulting in a cytoplasmic sequestration of the nuclear factor NF-κB, which is responsible for *iNos* gene upregulation [45]. Furthermore, cactus polysaccharides can prevent NO-synthase induced activity by oxygen and glucose deprivation in rat brain slices [46]. Our results suggest that CSO can also act in vivo against LPS-induced mouse brain *iNos*, possibly by preventing then the microglial-associated neuroinflammation [47].

The inhibitory effect of CSO on LPS-induced liver *Il-1β* and *Il-6* expression is in accordance with data reported by Kang et al. [48] and Aboura et al. [49] showing that extracts of *Ofi* seeds or *Ofi* cladode infusion attenuated *Il-1β* and *Il-6* expressions in high-fat diet-induced hepatic steatosis and inflammation, respectively. Interestingly, a study conducted by Attanzio et al. [50] in healthy human volunteers receiving a diet supplemented with cactus *Ofi* pear fruit pulp for 28 days revealed a reduction in pro-inflammatory markers, including *Il-1β*. Furthermore, other in vitro investigations underlined the anti-inflammatory effect of *Ofi* extracts on human chondrocyte [51] or murine macrophages [52].

As reported by Henry et al. [53], we showed that both the proinflammatory IL-1b and the anti-inflammatory IL-10 were induced by short-term LPS injection. CSO reduced the LPS-dependent expression of IL-1b (only in the liver) and IL-10. In addition, CSO induced slightly but significantly IL-4 expression in the brain. The IL-10 expression abrogates monocytes/macrophage-derived proinflammatory cytokines (i.e., TNF-α and IL-6) [54]. Accordingly, PPARδ and LXRs activations promote the deactivation of macrophage through increasing IL-10 production, resulting in the suppression of inflammation [55]. Accordingly, we previously reported that CSO extract can modulate microglial LXRs expression. This may illustrate the benefit related to CSO supplementation, particularly for the brain.

Another marked effect of CSO treatment (as OO or CO treatment) was the induction of the hepatic peroxisomal *Cat* gene. However, either OO or CO pretreatment downregulated *Cat* mRNA expression in the presence of LPS. Venkatesan et al. [56] reported that the induction of ROS downregulates catalase expression in mesangial cells through PI3 kinase/Akt signaling via the Forkhead box O1 transcription factor. Intriguingly, in our hands, this negative regulation depends on the concomitant administration of both OO (or CO) and LPS. By contrast, CSO pretreatment prevented such LPS-associated negative effects. On the other hand, the strong LPS induction of the hepatic peroxisomal Cu-Zn superoxide dismutase encoding gene, *Sod1*, was largely abrogated by either CSO, OO, or CO pretreatment. Although SOD represents the only family-enzyme able to specifically transform anion superoxide (O_2_^−^) into O_2_ and H_2_O_2_, several enzymes detoxify H_2_O_2_, including catalase, glutathione peroxidases, and peroxiredoxins [57,58]. Both *Cat* and *Sod1* genes are controlled transcriptionally by FOXO1 and Nrf2 transcription factors [59]. However, either *Cat* or *Sod1* mRNAs can be targeted by a specific miRNA, which may promote a differential regulation of their gene expressions [60]. Paradoxically, we showed that CSO, as well as OO or CO, treatment (or pretreatment in LPS-oil groups) can timely induce catalase activity or prevent its induction by LPS, respectively, in both brain and liver tissues. The modulation of catalase activity can also be explained by the posttranslational modifications of its polypeptide. Accordingly, it has been shown that CAT phosphorylation at Ser167 by protein kinase C delta [61] or at both Tyr231 and Tyr386 by Abelson tyrosine-protein kinases ABL1 and ABL2 [62] increases catalase activity, while CAT activity is decreased by nitrosylation of Cys377 [63] or S-thiolation [64].

The LPS downregulation of the *Acox1* gene expression was almost similar in brain and liver, respectively. However, either oil treatment alone or in the presence of LPS accentuates such downregulation in the brain. By contrast, CSO and CO pretreatment prevented such LPS-associated *Acox1* decreases in the liver. Of note, we have previously shown that the metabolic context may account for the differential cell response. Additionally, the preventive effect of certain oils, such as argan and olive oils, is dependent on the inflammatory status. LPS treatment leads, in a cytokines-dependent manner, to the increase of oxidative stress, the downregulation of peroxisome proliferator-activated receptor (PPAR) α activity, and peroxisomal dysfunction in developing rat oligodendrocytes [65]. In this context, the N-acetylcysteine, a strong antioxidant, restores PPARα activation and its peroxisomal target genes (i.e., *Abcd3* and *Acox1*) [65]. A decline in peroxisomal ACOX1 and CAT activities, involved in the β-oxidation and the antioxidative pathways, respectively, has been reported during aging [11,66]. The key role of peroxisomal function in aging, and related inflammation processes, is conserved from single-eukaryotic cells to higher vertebrates such as humans [67]. In addition, peroxisomes have recently been described as pivotal players in the regulation of immune functions and inflammation during development and infection [67]. Thus, preserving peroxisomal functions by CSO supplementation could also protect against inflammation and oxidative stress. The recovery of the *Acox1* gene expression by CSO in LPS-treated mice could be attributed to the remarkable composition of CSO in tocopherols and in PUFA, which are present at high levels in CSO when compared to OO and argan oil [23,68]. The *Acox1* induction by PPARα-dependent PUFA activation has been thoroughly documented [11,69]. Accordingly, we have previously demonstrated that argan oil regulates liver fatty acid oxidation pathways through the activation of the nuclear receptors PPARα, ERRα, and their coactivator, PGC-1α [70]. Interestingly, the LXR nuclear receptors, designed as integrators of metabolic and inflammatory signaling [71], can be modulated by CSO phytosterols [29]. Furthermore, sitosterol, the main phytosterol present in CSO or OO, elicited an anti-inflammatory effect through the downregulation of several components of the TLR4 pathway [72].

## 4. Conclusions

Collectively, in the present study we showed that CSO possesses protective effects against short-term LPS-induced brain and liver metabolic stress by restoring the peroxisomal antioxidant and fatty acid β-oxidation functions. Thus, the CSO hepato-protective response is efficient in the short term (i.e., 4 h) by restoring hepatic peroxisomal antioxidant and β-oxidative capacities. For the first time, we identified that CSO also has a neuroprotective effect against sepsis, maintaining the peroxisomal antioxidant enzyme activities of catalase and GPx. In the future, a combination of lipidomic and transcriptomic analysis would clarify the metabolic signaling pathways involved in the CSO neuro- and hepato-protective actions against LPS. Additionally, it would be interesting to explore the potential effects of individual CSO components, such as tocopherol and polyphenol derivatives. This may document the potential beneficial role of CSO in lowering the deleterious effects of sepsis and as a new therapeutic option with less adverse effects than synthetic compounds.

## 5. Material and Methods

### 5.1. Chemicals and Reagents

RNeasy Mini kit and QIAzol reagent (Qiagen, Courtaboeuf, France); iScript cDNA Synthesis Kit (Bio-Rad, Marnes-la-Coquette, France); Takyon ROX SYBR 2X MasterMix dTTP blue (UF-RSMT-B0701, Eurogentec, Angers, France); Pierce™ BCA kit (Thermo Fisher Scientific, Illkirch, France). Applied Biosystem Step One QPCR machine (Thermo Fischer Scientific, Illkirch, France), Potter Elvehjem homogenizer (Dominique Dutscher, Issy-les-Moulineaux, France), Anti-ACOX1 (BioPeroxIL laboratory, Dijon, France), and anti-catalase (ab76024, Abcam, Paris, France). SuperSignal™ West Femto Maximum Sensitivity Substrate (ECL) Solutions (Thermo Fisher Scientific, Illkirch, France). Other chemicals were purchased from Sig-ma-Aldrich (Saint-Quentin-Fallavier, France).

### 5.2. Origin, Extraction, and Composition of Oils

*Cactus seed oil preparation*: prickly pear fruits were obtained from the Cooperative of Sabbar Rhamna (Skhour Rhamna, Morocco). Seeds and juice were separated by an industrial prickly pear juice extracting machine (Philips Viva HR1832/00, Mumbai, India). Juice was stored for another use at −20 °C after measuring its pH, whereas seeds were washed thoroughly with water, air dried, and then used to extract seed oil by using a cold-press machine (Longer machinery, LGYL-80A, Henan, China). CSO, obtained from Driss Mistahi, was stored in the darkness. Olive oil (OO) (Olea europaea L. cv. Moroccan picholine) was obtained from the Aklim region, latitude: 34°55′45″ N; longitude: 2°26′7″ W, Berkane, Morocco. Colza oil (CO) (Brassica napus subsp. Napus) was obtained from a commercial supermarket.

### 5.3. Mice Treatments

C57BL/6 J male mice (12–16 weeks old) were purchased from Pasteur medical Laboratory in Casablanca, Morocco. Mice were used under the recommendations of the Organization for Economic Co-Operation and Development (OECD). All animal experiments were carried out according to ethical rules of the University of Hassan I and according to the National Institutes of Health guide for the care and use of Laboratory Animals (NIH publication No. 85-23, revised 1985). All mice were housed under light-dark (12 h–12 h) cycles, relative humidity (45–65%), at a temperature of 22 ± 2 °C, and fed with standard diet and water ad libitum. Three weeks after acclimatization, the mice were randomly divided to eight groups (5 mice/group), each group receiving for 28 days a standard diet added or not with a vegetal oil: 2 control groups fed with a standard diet; 2 cactus seed oil groups fed with a standard diet supplemented with 6% (*w*/*w*) CSO; 2 olive oil groups fed with a standard diet supplemented with 6% (*w*/*w*) OO; and 2 colza oil groups who received a standard diet supplemented with 6% (*w*/*w*) CO. We solubilized each oil in acetone (1:4 *v*/*v*). This mixture was added to diet pellets and then evaporated overnight. Four hours before euthanasia and during the fed state, one group from the two groups (control (+LPS), AO (AO + LPS); OO (OO + LPS) and CO (CO + LPS)), received an injection (5 mg/kg) via tail vein of 100 µg of LPS from Escherichia coli O111:B4 (Sigma, Saint-Quentin-Fallavier, France) prepared in sterile phosphate-buffered saline (PBS), or an equal volume of PBS alone [18]. The LPS serotype of Escherichia coli O111:B4 has been already used in a short-term treatment of C57BL/6 J mice according to other and our previous studies [18,70,73,74]. Brain and liver tissues were harvested immediately after euthanasia and frozen in an ethanol-dry ice bath and stored at −80 °C.

### 5.4. Measurement of Enzymatic Activities

Catalase and GPx activities ware measured as described by Essadek et al. (2022). Peroxisomal acyl-CoA oxidase (ACOX1) activity was estimated by the fluorometric assay using palmitoyl-CoA as a substrate as described previously [43].

### 5.5. Evaluation of Gene Expression by Quantitative RT-qPCR

We used the RNeasy Mini kit (Qiagen, Courtaboeuf, France) to isolate total RNA from brain and liver tissues by following the manufacturer’s instructions. The concentration of total RNA was performed by spectrophotometry at 260 nm using a TrayCell (Hellma, Paris, France). An amount of 100 ng of total RNA was used for the reverse transcription reaction to generate cDNA by the iScript cDNA Synthesis Kit (Bio-Rad). Quantitative PCR analysis for each specific gene was performed in triplicate, using the Takyon ROX SYBR 2X MasterMix dTTP blue (Eurogentec, Angers, France), on an Applied Biosystem Step One QPCR machine (Life Science Technologies, Saint-Aubin, France). The primers sequences are given in Table 1. Cycling conditions were as the following: activation of DNA polymerase at 95 °C for 10 min, followed by 40 cycles of amplification at 95 °C for 15 s, 60 °C for 30 s, and 72 °C for 30 s. A melting curve analysis was performed at the end of each reaction to test the absence of non-specific products. The quantification of gene expression was calculated using cycle threshold (Ct) values and standardized by the 36B4 reference gene. The relative expression of genes was determined by the 2^−ΔΔCt^ method. Results are shown as graphs of relative expression data (fold induction) with fold positive values representing the up-regulation, fold negative values the down-regulation, and 0 as no variation of the expression [75].

### 5.6. Immunoblotting

The lysis of mice tissues (brain or liver) was accomplished in 4% (*w*/*v*) or 10% (*w*/*v*) RIPA buffer (50 mM Tris-HCl, pH 8.0, 150 mM NaCl, 1% NP-40, 0.1% SDS, 0.5% sodium deoxycholate), using a potter Elvehjem homogenizer (Dominique Deutscher, Issy-les-Moulineaux, France). The obtained lysates were centrifuged at 10,000× *g* for 10 min at 4 °C, and the supernatants were used for protein content measurement by the Bicinchoninic Acid Solution (ThermoFisher Scientific, Illkirch-Graffenstaden, France). We diluted fifty µg of proteins (*v*/*v*) in the loading buffer (125 mM Tris-HCl, pH 6.8, 4% SDS, 20% glycerol, 14% mercaptoethanol, and 0.003% Bromophenol blue) and heated samples at 96 °C for 5 min, then they were separated on a 10% or 12% SDS-PAGE and transferred onto PVDF membrane. The non-specific binding sites were blocked with 5% nonfat milk in TBST (10 mM Tris-HCl, 150 mM NaCl, 0.1% Tween 20, pH 8) for 1 h at room temperature. Incubation of the membrane with the primary antibody diluted in 1% milk TBST was performed over-night at 4 °C (anti-catalase, (ab76024, Abcam, Paris, France), dilution 1/2000; anti-β-actin, (A544, Sigma-Aldrich, Saint-Quentin-Fallavier, France), dilution 1/5000; anti-ACOX1 was made by BioPeroxIL laboratory (Dijon, France), dilution 1/200). The membranes were washed 3 times for 10 min in PBST and incubated for 1 h with a secondary appropriate horseradish peroxidase-conjugated antibody diluted in 1% milk TBST (dilution 1/10,000) at room temperature. After three washes in TPBS for 10 min, the bands were developed by chemiluminescence using the Supersignal West Femto Maximum Sensitivity Substrate (ThermoFisher Scientific, Illkirch-Graffenstaden, France) and a Chemidoc XRS+ device (Bio-Rad, Marnes-la-Coquette, France). The Image Lab software (Bio-Rad) was used for quantification.

### 5.7. Statistics

All experimental values are expressed as the average of mean ± standard deviation. The error bars presented on the figures correspond to the standard deviation. Statistic significances were calculated by two-way ANOVA and Tukey’s multiple comparisons test, with a significance level of *p* ≤ 0.05.

## Figures and Tables

**Table 1:** Sequences of the primers used for qPCR.

Gene Name	Primer Sequences
*Acox1-F* *Acox1-R*	5′TCGAAGCCAGCGTTACGAG3′ 5′GGTCTGCGATGCCAAATTCC3′
*Catalase-F* *Catalase-R*	5′AGCGACCAGATGAAGCAGTG3′ 5′TCCGCTCTCTGTCAAAGTGTG3′
*Il-1β-F* *Il-1β-R*	5′GAGATTGAGCTGTCTGCTCA 3′ 5′AAGGAGAACCAAGCAACGAC 3′
*Il-4-F* *IL-4-R*	5′CCATATCCACGGATGCGACAA3′ 5′CCTCGTTCAAAATGCCGATGAT3′
*Il-6-F* *Il-6-R*	5′GTTCTCTGGGAAATCGTGGA3′ 5′TGTACTCCAGGTAGCTATGG3′
*Il-10-F* *Il-10-R*	5′GCTGGACAACATACTGCTAACC3′ 5′CCCAAGTAACCCTTAAAGTCCTG3′
*iNos-F* *iNos-R*	5′CCTAGTCAACTGCAAGAGAA3′ 5′TTTCAGGTCACTTTGGTAGG3′
*Sod1-F* *Sod1-R*	5′AACCAGTTGTGTTGTCAGGAC3′ 5′CCACCATGTTTCTTAGAGTGAGG3′
*36b4-F* *36b4-R*	5′CGACCTGGAAGTCCAACTAC3′ 5′ATCTGCTGCATCTGCTTG3′
